# A dataset quantifying polypharmacy in the United States

**DOI:** 10.1038/sdata.2017.167

**Published:** 2017-10-31

**Authors:** Katie J. Quinn, Nigam H. Shah

**Affiliations:** 1 Stanford Center for Biomedical Informatics Research, Stanford, California, CA 94305, USA

**Keywords:** Data integration, Data mining, Drug therapy, Pharmacology

## Abstract

Polypharmacy is increasingly common in the United States, and contributes to the substantial burden of drug-related morbidity. Yet real-world polypharmacy patterns remain poorly characterized. We have counted the incidence of multi-drug combinations observed in four billion patient-months of outpatient prescription drug claims from 2007–2014 in the Truven Health MarketScan® Databases. Prescriptions are grouped into discrete windows of concomitant drug exposure, which are used to count exposure incidences for combinations of up to five drug ingredients or ATC drug classes. Among patients taking any prescription drug, half are exposed to two or more drugs, and 5% are exposed to 8 or more. The most common multi-drug combinations treat manifestations of metabolic syndrome. Patients are exposed to unique drug combinations in 10% of all exposure windows. Our analysis of multi-drug exposure incidences provides a detailed summary of polypharmacy in a large US cohort, which can prioritize common drug combinations for future safety and efficacy studies.

## Background and Summary

Concomitant use of multiple prescription drugs (‘polypharmacy‘) is increasingly common, with 10% of the population^1,2^ and 30% of older adults in the United States taking five or more drugs simultaneously^1–3^. Similarly high prevalence is reported in other countries (e.g., the United Kingdom^4^, Sweden^5^, China^6^, Brazil^7^, and India^8^. The prevalence of polypharmacy is driven by high rates of comorbidities (in the United States in 2012, 26% of all adults, and 61% of adults over 65 years of age had two or more chronic conditions^9^), and exacerbated by clinical practices enabling overprescription and insufficient monitoring^10,11^. Drug-related morbidity has become a substantial healthcare burden: in the United States, adverse drug reactions are prevalent (causing 4 hospitalizations per 1000 people each year^10^), serious (among top 10 common causes of death^12^), and expensive (with associated annual costs estimated at US$30billion^13^ to US$180billion^14^).

Exposure to multiple drugs puts patients at additive risk of each single drug’s potential adverse outcomes. In a study of an elderly cohort, the strongest predictor of a potentially harmful medication was the number of drug prescriptions^15^. But drugs can also interact to increase risk beyond ‘the sum of the parts’, either by canceling an intended drug action, enhancing existing risks, or creating new risks. It’s estimated that over 20% of adverse drug reactions are due to underlying drug interactions^16,17^, and that risk of drug interaction increases with the number of drugs taken^18^. However, despite increasing awareness of morbidity related to polypharmacy, multi-drug exposure patterns remain poorly characterized.

Insurance claims records enable analysis of prescription practices in a large patient cohort, even for drug regimens that would be rare in smaller cohorts. The 21st Century Cures Act, enacted in December 2016, recognizes the value of, and mandates the use of, observational patient-experience data, such as insurance claims, for drug surveillance^19^. The relative strengths of insurance claims for characterizing population-level drug use are that data reflect prescriptions that are actually dispensed to the patient, and capture prescription information across very large cohorts. As for most sources of drug use data, whether patients actually ingested the drugs remains a limitation.

**Figure 1:**
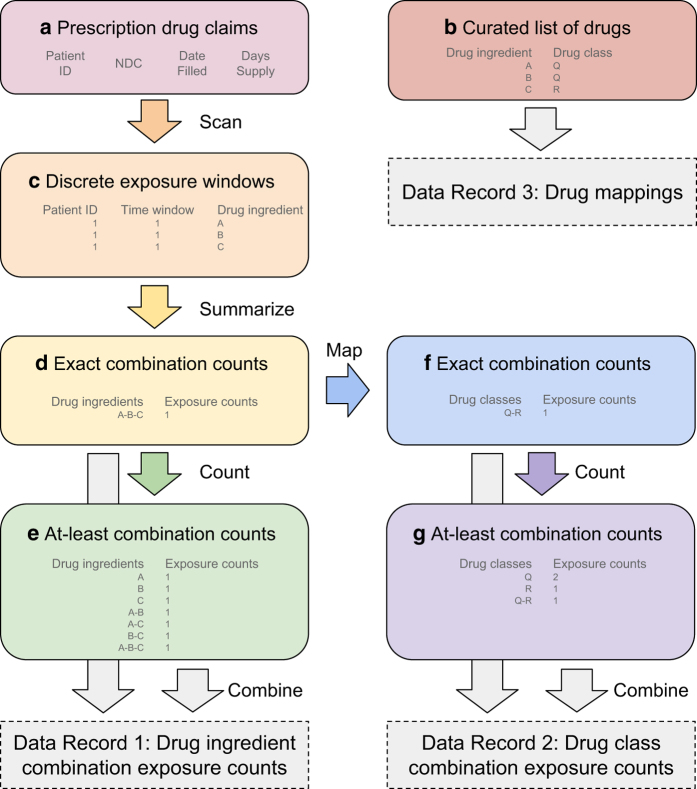
Data analysis workflow to generate drug combination exposure incidences from prescription drug claims. Prescription drug claims (**a**) are scanned to create discrete exposure windows (**c**) for the set of drugs **(b**). These windows are summarized to produce ‘exact’ exposure incidences at the drug ingredient level (**d**). This table is the substrate for counting the incidence of exposure to ‘at least’ drug combinations (**e).** Exposure counts for combinations of *N*=1 to 5 drug ingredients are published in Data Record 1. Exact drug ingredient combinations (**d**) are translated to drug class combinations (**f**), keeping only unique classes. Again, these are used to count the exposure incidence of ‘at least’ drug class combinations (**g**). Exposure counts for combinations of *N*=1 to 5 drug classes are published in Data Record 2.

Here, we publish a dataset of multi-drug exposure incidence in a large insured cohort in the United States, both in terms of drug ingredients and drug classes. We analyze outpatient prescription drug claims from the Truven Health MarketScan® Research Databases, which contain health coverage records for over 100 million employees, dependents, and retirees in the United States from 2007–2014, amounting to over 4 billion months of patient observation. Table 1 summarizes relevant metrics of the database. Prior to our work, Sutherland *et al.* have reported on co-prescription trends using self-reported data from a small but nationally-representative cohort of 10,000 NHANES participants. The frequency of some drug-pair exposures among elderly participants was also reported^2^. To our knowledge, ours is the first study to quantify the incidence of specific combinations of more than two drugs. Figure 1 summarizes our workflow of processing prescription drug claims into discrete exposure time-windows, and then counting concomitant drug exposures for drug ingredients and ATC-II drug classes.

This dataset will benefit researchers who study multi-drug safety or efficacy. The most common multi-drug combinations can be prioritized for subsequent studies of multi-drug safety or efficacy. As a side benefit, by mapping drugs to disease based on indications, the dataset can also provide a summary of comorbidities that drive the observed prescription trends.

This dataset will also benefit practitioners by enabling risk stratification of patients based on the multi-drug combinations they are on; for example, the dataset enables analyses identifying associations of specific drug combinations with health outcomes (such as emergency department visits), which could enable patient risk stratification at the time of medication reconciliation.

Ultimately combined analyses spanning both safety and risk stratification will enable systematic progress towards safe polypharmacy. With roughly 40 million individuals experiencing polypharmacy in the US and as many as 10% of all adults worldwide, the existence of such datasets is crucial for a data-driven quantification of which drug combinations are risky.

## Methods

### Data source

Prescription drug claim data were derived from the Truven Health MarketScan 2007 to 2014 Commercial Claims and Encounters and Medicare Supplemental and Coordination of Benefits Databases, which were accessed via the Stanford Center for Population Health Sciences Data Core. Further details about the Data Core and its operating procedures are available at http://med.stanford.edu/phs/phs-data-center.html. These databases represent the health services of approximately 100 million employees, dependents, and retirees in the United States with primary or Medicare supplemental coverage through privately insured fee-for-service, point-of-service, or capitated health plans. The Commercial Claims and Encounters, and Medicare Supplemental and Coordination of Benefits populations comprise 90 and 10% of the total cohort respectively, with a mean age of 33 and 73 years and gender fraction of 49 and 45% male, creating a combined cohort with a mean age of 37 years and 48% male. Patients are observed for a median of 29 months. We analyzed the outpatient prescription drug claims of 150 million patients over 4.3 billion months of patient enrollment. We focus on outpatient prescriptions since 90% of all prescriptions are in the outpatient setting^20^, and inpatient drug treatment patterns differ substantially.

### Drug list curation and drug mapping

We curated a set of 1429 drugs, defined by RxNORM ingredient level, by beginning with the 1165 drug ingredients occurring in all of DrugBank, RxNORM, and UMLS (previously curated in our lab^21^), adding drug ingredients occurring commonly in the Truven Health MarketScan Databases, and removing vaccines, and vitamins and minerals (which are more often obtained over-the-counter than by prescription).

Of this set of 1429 drug ingredients, 864 occur in the Truven Health MarketScan Database prescription claims. Drugs are identified in prescriptions using National Drug Codes (NDCs). We built a mapping of NDCs to RxNORM-defined drug ingredients using the NLM’s RxMix API to match on strings containing drug names, first with strict matching (which maps the majority of NDCs with very low error), then with approximate string matching (which maps the remaining NDCs but requires manual validation of string matches).

Combination drugs (e.g., Norco) count as exposure to each drug ingredient (e.g., acetaminophen and hydrocodone). The approximate drug cost per day was calculated as the median payment-per-days-supply for all patient orders of that drug. Drugs are also classified by Anatomical Therapeutic Chemical (ATC) class at the second level (‘therapeutic main group’), by mapping RxNORM identifiers to ATC codes. The 864 unique drug ingredients in the dataset map to 79 unique second-level ATC classes.

### Extracting discrete exposure windows from drug prescriptions

**Figure 2:**
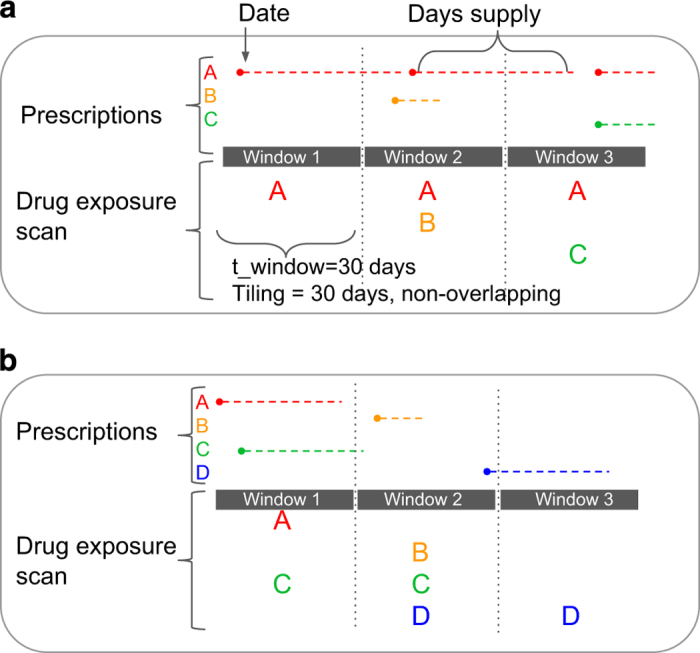
Illustration of conversion of drug prescription date of service and days of supply into discrete exposures. (**a**) Shows three typical prescription patterns, converted to exposure in three windows, using non-overlapping 30-day windows. (**b**) Shows uncommon prescription patterns that introduce error in interpretation of concomitant exposure: While A and B are separated by only a few days, and may be considered concomitant, they are not counted as concomitant exposures; While Drugs C and D are separated by many days, they are recorded as concomitant exposures in Window 2.

To count concomitant drug exposures from prescription claims, we first scanned drug prescriptions into discrete exposure windows. We defined exposure periods as non-overlapping 30-day windows. We selected a 30-day window because it is the most common prescription duration, and thus a natural timescale for prescriptions. A patient is considered exposed to a drug starting from the date the prescription and for the duration of the days-of-supply. If any of those days overlap with a window, the patient is considered exposed in that window (Fig. 2a). This method is computationally efficient and provides a good approximation of concomitant exposure.

As a known limitation, the method overestimates exposures, and thus concomitant exposures, if a patient does not take the prescription for its full duration. As shown in Fig. 2b, non-overlapping windows introduce error when either: 1) non-concomitant prescriptions are separated by less than 30- days yet both overlap with a particular exposure window; or conversely 2) when prescriptions separated by only a few days fall into different exposure windows. We create exposure windows using a simple integer division of patient age-in-days by 30, which is computationally efficient. However this creates partial windows of observation at the beginning and end of each patient’s eligibility period, with a mean duration of 15-days. Given that patients are observed for a median of 29 months, this error is present only in about 5% of windows. However, these 30-day non-overlapping windows simplify computation, with a low error rate, for the purposes of ranking the most common multi-drug exposures.

Using this method, individual patient prescription claims were converted into drug exposures in discrete windows (Fig. 1c), resulting in 5.1 billion drug exposures. This dataset was then used to count concomitant drug exposure.

### Counting concomitant multi-drug exposures

There are two ways to count multi-drug exposure: exposure to an ‘exact’ set of drugs (and no additional drugs), and exposure to ‘at least’ a particular set of N-drugs (which may or may not be taken with additional drugs). Each of these variants captures valuable information: ‘exact’ counts quantify the absolute number of concomitant drug exposures, and how many patients are exposed to a precise sets of drugs; ‘at least’ counts are important for knowing all patients exposed to any given drug combination. See example shown in Fig. 1: Concomitant exposure to drug ingredients A (class Q), B (class Q), and C (class R) will contribute a count to A+B+C for ‘exact’ drug ingredient exposure, and each of A, B, C, A+B, A+C, B+C, and A+B+C for ‘at least’ drug exposure.

**Figure 3:**
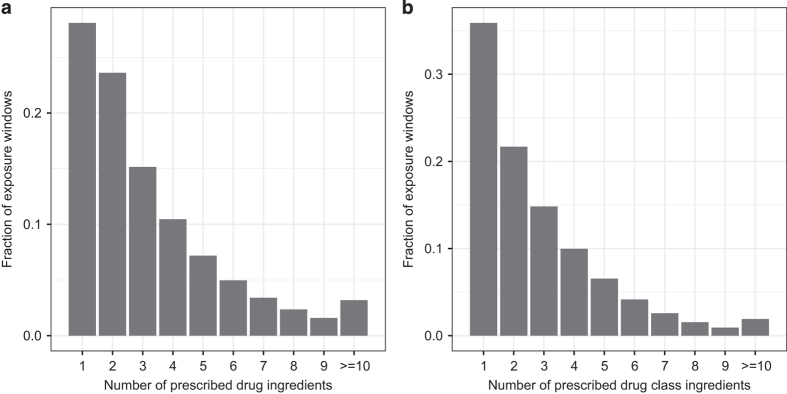
Distributions of the number of unique concomitant drug exposures per patient-months. Distributions are for concomitant exposures to (**a**) drug ingredients and (**b**) drug classes, truncated at 10, across the 3.0 billion observed patient-months, including 1.7 billion with prescription drug exposures. (The 43% (=1.3/3 billion) of patient-months with no drug exposures are not shown on these plots.) Patients taking any prescription drugs are exposed to a median of 2 and 95th-percentile of 8 drug ingredients, and a median of 2 and 95th-percentile of 7 unique drug classes.

This method counted 220 million unique ‘exact’ drug combinations exposures, with patients exposed to a median of 2 drugs and 95th-percentile of 8 drugs per window (Fig. 3a). In approximately 10% of windows, patients were exposed to a unique set of drugs, never observed elsewhere in the entire database. This is in agreement with a recent study of treatment pathways that found that 10% of diabetes and depression patients and almost 25% of hypertension patients received therapeutic regimens that were unique within a 250-million-large patient cohort^22^.

To count ‘at least’ multi-drug exposures, we created a drug-based index to the summarized 220 million ‘exact’ counts. (This required much less computation than indexing on the original 5.1 billion exposure windows.) We then performed an intersect operation for each ‘at-least’ drug combination of interest. Counting all possible drug combinations is infeasible, and unnecessary since most combinations are never observed. The challenge was to create a list of N-drug combinations likely to have high concomitant exposures. We achieved this with a ‘greedy’ approach of constructing N-drug combinations from N-minus-1 subset drug combinations observed in at least 1000 exposure windows, for each of N=2, 3, 4 and 5 drugs.

An additional metric of interest is the extent to which drug combinations are concomitant beyond what would be expected by chance, given their marginal frequencies. Drug combinations’ overrepresentation was defined as the ratio of the observed-to-expected drug combination incidence in two ways: first (for N>1) based on single-drug frequencies, which gives the overall overrepresentation; and second (for N>2) based on the minimum of each of N permutations of (N-1)+1 drug subsets, which is greater than the single-drug overrepresentation, and gives the overrepresentation of the drug combination beyond its subsets. (As an example, the co-incidence of drugs A+B+C is compared to the incidence expected by chance based on the incidences of drugs A+B and C, A+B and B, and B+C and A. The smallest overrepresentation is reported. The second method is only reported for N>2, because the two methods are equivalent for N=2.)

We repeated these computations of ‘exact’ and ‘at least’ exposure counts, and their overrepresentation, for the 79 second-level ATC drug classes. Second-level ATC drug class names were extracted from the website of the WHO Collaborating Centre for Drug Statistics Methodology. Though one drug ingredient can map to multiple ATC classes, we count only the primary class. Continuing the example in Fig. 1, concomitant exposure to drugs from classes Q and R would be counted as (iii) Q+R for ‘exact’ drug class exposure, and (iv) Q, R, and Q+R for ‘at least’ drug class exposure. (Note that drug classes are counted only once, even if a patient is taking two or more drugs from a particular class). This calculation yielded 39 million unique exact drug class exposures, with patients exposed to a median of 2 and 95th-percentile of 7 drug classes per window (Fig. 3b).

### Code availability

Code used to generate the dataset is available on a public github repository (https://github.com/katieq/QuantifyingPolypharmacy). To avoid disclosing the format of the Truven Health Marketscan Databases, the code begins at the step of analyzing prescription data extracted into a data-frame with columns for a patient identifier, drug identifier, age of prescription, and days of supply.

## Data Records

The dataset of exposure counts for drug and drug-class combinations is publicly available online at Dryad (Data Citation 1) in 12 tab-delimited data files and a README.txt file. The tab-delimited data files are outlined below and in Table 2. The accompanying README.txt file contains filenames and descriptions of file contents. Data files can be accessed directly by their associated URLs, for example by reading into R with the readr package’s read_tsv function. Table 2 summarizes attributes of the underlying patient claims data in the 2007–2014 Truven Health MarketScan Commercial and Medicare Supplemental Databases.

### Data Record 1: Drug ingredient combination exposure counts

Data Record 1 contains the exposure incidences for the most common combinations of *N=1-to-5* drugs in five files, with one row per combination. All single drugs (N=1) and drug pairs (N=2) are included; for N=3-to-5, drug combinations with at least 10,000 exposure counts are included. Exposure counts below 100 patient-windows are reported as ‘<100’ to protect patient privacy. Each row contains *N+5* tab-delimited columns comprising: the name for each drug ingredient, the count of windows with concomitant exposure to this drug combination, potentially concomitant with additional drugs (*atleast_exposure_count*), the count of windows with concomitant exposure to this drug combination and no additional drugs (*exact_exposure_count*), the ratio of the two previous columns (*fraction_exact*), the ratio of the *atleast_exposure_count* to the total number of observed windows with any prescription (*fraction_all_windows*), overrepresentation beyond expected based on marginal frequencies of single drugs (*observe_per_expect_1s*) and (N-1)+1 drug subsets (*observe_per_expect_N1*), and an estimate of the daily cost of the drug combination (*estimate_drug_combo_cost_per_day*).

### Data Record 2: Drug class combination exposure counts

The contents of Data Record 2 are equivalent to Data Record 1, but for level-II ATC drug classes instead of drug ingredients. The record also contains five files for combinations of *N=1-to-5* drug classes, with one row per combination. As for Data Record 1, all single drug class (N=1) and drug class pairs (N=2) are included, and drug class combinations with at least 10,000 exposure counts are included for N=3-to-5; exact exposure counts of less than 100 are reported as ‘>100’. Columns are equivalent to Data Record 1’s, with drug class names replacing drug ingredient names. However daily cost can not be calculated at the drug class level.

### Data Record 3: Drug mappings

Data Record 3 contains two tab-delimited files containing the list of 1429 drug ingredients and 93 corresponding ATC level-2 drug classes considered in this study. The drug ingredient file contains one drug per row sorted alphabetically by drug ingredient name, with five columns for the drug ingredient name, RxNorm CUI number, UMLS CUI, Drug Bank ID, ATC code, second-level ATC drug class name, and estimated median cost per day. The drug class mappings file contains one ATC level-2 drug class per row sorted alpha-numerically by ATC class, with two columns for the ATC code and name. (Thus the ATC level-2 class names in the drug ingredient file are redundant, but included for convenience).

## Technical Validation

We validated our method in three ways. First, we compared our computational method’s results to manual counting, by reading the dates and days-supply for a random sample of ten patients’ 260 drug prescriptions. The counts of concomitant drug exposures matched perfectly, indicating that our method does indeed accurately extract concomitant drug exposures as intended without errors in arithmetic.

Second, we conducted a sensitivity analysis on the duration of the discrete exposure window, by counting concomitant drug exposures in a random sample of 10,000 patients with an exposure window of 10, 20, 30, 40, 50, 60, and 90 days. Since all prescriptions are considered ‘exposures’ for the entire duration of the window, longer windows slightly increase the mean drug exposure counts (average drug exposure count is 3.1, 3.2 and 3.8 for a 10-, 30-, or 90-day window respectively), and thus increase the relative incidence (i.e., ranking) of prescriptions with short-durations (e.g., antibiotics or short-term pain relief). Thus the choice of exposure window duration *does* affect the Data Records. Therefore we set the window duration equal to the most common prescription duration. Prescriptions in this claims database are most often for 30 days (50% of all prescriptions), with about 20% for 10 or fewer days. Thus a 30-day window is an appropriate timescale to capture changes in drug exposures.

Finally, we tested the sensitivity to cohort size, by comparing the drug combination incidence ranking obtained using the entire Truven Health MarketScan cohort (approximately 100 million patients) to a random sample of 100,000 (1e5) patients, and 1,000,000 (1e6) patients. As expected, analysis of smaller cohorts obtains a similar *ranking* of the common drug combinations, but inaccurately estimates the incidence, and thus the ranking, of rare drug combinations. In addition, smaller cohorts overestimate the patients exposed to unique drug combinations, never observed elsewhere in the database: In the complete cohort, 10% of drug combinations are observed only once, but in a cohort of 1e5 patients, that fraction is 20%. Thus while smaller cohorts are sufficient to rank the incidence of common drug combinations, a large patient cohort is required to accurately estimate the incidence of drug combinations.

### Limitations

The accuracy of this dataset as a summary of multi-drug exposure incidences in the United States is limited to some extent by the underlying data source and our method of computation. The Truven Health MarketScan Research Databases cohort is commercial claims, and not a fully representative sample of the United States population. We examine drug exposure based on filled prescriptions, but patients may take none or only a fraction of the dispensed drugs. Since there is bias on adherence *between* drugs, this will introduce bias in the resulting single drug and drug combination incidences. However, billing data from filled prescriptions are more accurate than alternative sources, such as doctor’s notes or prescription orders that may go unfilled.

We only observe and analyze prescription drugs, but over-the-counter drugs and supplements contribute a significant portion of total drug exposures^23^. Though patient surveys can offer information about exposure to over-the-counter drugs and supplements, they rely on patient memory, and lack the cohort size and accuracy of prescription records.

Our method scans prescription drug claims according to 30-day exposure windows. Shorter or longer exposure windows would increase or decrease apparent multi-drug exposures respectively. The size of the exposure window affects drug combinations’ relative incidence (i.e., ranking), with longer windows increasing the apparent incidence of combinations including drugs with short prescription durations (e.g., antibiotics or short-term pain relief). However the rankings are agnostic to exposure window duration with our choice of a window of 30-days, to match the days-of-supply of the majority of prescriptions.

Finally, our analysis uses all data from 2007–2014, ignoring the likely non-stationarity of prescription patterns^24^ (as suggested by the increase in prevalence of polypharmacy from 8% to 15% between the 1999–2000 and 2011–2012 NHANES surveys^23,25^). Nonetheless, our multi-drug exposure dataset provides a ranking of common concomitant prescription drug exposures for a large population in the United States.

## Usage Notes

This summary of multi-drug prescription patterns in a large cohort enables further analysis of the trends, safety, or efficacy of multi-drug use.

### Prioritize common multi-drug combinations for adverse event association analysis

The common multi-drug combinations identified here can now be prioritized for analysis of association with adverse health outcomes. An example illustrating this use case is identifying which of the common 3-drug combinations in Data Record 1 are most overrepresented in the 30-days prior to Emergency Department visits (Table 3). It is important to note that this association tells us nothing about causation, but merely identifies drug combinations taken at increased rates by patients prior to ED visits. Thus, as indicators of patients’ health state, multi-drug combinations could potentially be used to identify patients at risk of an ED visit in the near-future. Similar association analysis can be completed with any desirable or undesirable outcome, in any cohort of interest, for various study designs.

### Identify drugs used concomitantly with a given drug of interest

This dataset can be used to profile the common co-exposures for any drug ingredient or class of interest. Table 4 shows this analysis for the first line diabetes drug metformin and the opioid oxycodone. The summary was obtained by extracting rows containing metformin or oxycodone from the 2-drug table of Data Record 1, and normalizing by the total exposure counts for metformin or oxycodone. Code to perform this analysis for any drug ingredient or drug class of interest is provided in the R-script *get_codrugs.R* at the code repository (https://github.com/katieq/QuantifyingPolypharmacy). This could be repeated for larger drug combinations using the 3-, 4-, or 5-drug tables.

### Stratify patients by risk of adverse health outcomes, based on prescription set

This dataset can now be used to calculate the increased risk of undesirable health outcomes associated with a particular set of prescriptions. Such a risk estimate can be used to stratify patients according to risk of future adverse health events, and then to flag prescription changes that place patients in a higher risk category, or to identify prescription combination changes that lower patients’ risk category. Of course, such risk stratification implies no causality whatsoever; however, such analyses can provide a succinct report on the risks experienced by a cohort of similarly-treated patients.

## Additional information

**How to cite this article:** Quinn, K. J. & Shah, N. H. A dataset quantifying polypharmacy in the United States. *Sci. Data* 4:170167 doi: 10.1038/sdata.2017.167 (2017).

**Publisher’s note:** Springer Nature remains neutral with regard to jurisdictional claims in published maps and institutional affiliations.

## Supplementary Material



## Figures and Tables

**Table 1:** Summary of Truven Health MarketScan Research Database prescription data and drug combination counts.

**Prescription claims database summary statistics:**	
Number of patients	82 million
Median months of patient observation	30
Range of months of patient observation (10% to 90%)	8 to 84
Number of months of patient observation	3.0 billion
Number of months with drug exposures	1.7 billion
Fraction of all months of patient eligibility with any drug exposures	57%
Total number of prescription drug claims	3.2 billion
*Drug combination counting summary statistics*:	
Number of discrete 30-day window drug exposures	5.1 billion
Number of unique drug ingredient combinations	220 million
Fraction of windows with unique drug ingredient exposure	10%
Number unique drug class combinations	39 million
Fraction of windows with unique drug class exposure	2%

**Table 2:** Data Records description.

	**Description**	**File or column name**
	Data Record 1: Drug ingredient combination exposure counts	
*Files*	5 files, for combinations of N=1-to-5 drug ingredients	*db_drugs_Ns.tsv*
*Columns*	Drug ingredient name (N columns)	*drug_name_A*
	Count of windows with concomitant exposure to this drug combination: potentially concomitant with additional drugs	*atleast_exposure_count*
	Count of windows with concomitant exposure to this drug combination and no additional drugs	*exact_exposure_count*
	Estimate of the daily cost of the drug combination	*estimate_drug_cost_per_day*
	Fraction of exposure counts that occur with no additional drugs (equal to the ratio of the exact to at-least exposure counts)	*fraction_exact*
	Ratio of the *atleast_exposure_count* to the total number of observed windows with any prescription	*fraction_all_windows*
	Ratio of the combination’s observed to expected incidence (*atleast_exposure_count*) based on marginal frequencies of single drugs (applicable for N>1).	*observe_per_expect_1s*
	Ratio of the combination’s observed to expected incidence (*atleast_exposure_count*) based on marginal frequencies of (N-1)+1 subsets (applicable for N>2).	*observe_per_expect_N1*
	Data Record 2: Drug class combination exposure counts	
*Files*	5 files, for combinations of N=1-to-5 drug classes	*db_atc_classes_Ns.tsv*
*Columns*	Drug class code (N columns)	*atc_code_A*
	Drug class name (N columns)	*atc_name_A*
	Count of windows with concomitant exposure to this drug combination: potentially concomitant with additional drugs	*atleast_exposure_count*
	Count of windows with concomitant exposure to this drug combination and no additional drugs	*exact_exposure_count*
	Fraction of exposure counts that occur with no additional drugs (equal to the ratio of the exact to at-least exposure counts)	*fraction_exact*
	Ratio of the *atleast_exposure_count* to the total number of observed windows with any prescription	*fraction_all_windows*
	Ratio of the combination’s observed to expected incidence (*atleast_exposure_count*) based on marginal frequencies of single drugs (applicable for N>1).	*observe_per_expect_1s*
	Ratio of the combination’s observed to expected incidence (*atleast_exposure_count*) based on marginal frequencies of (N-1)+1 subsets (applicable for N>2).	*observe_per_expect_N1*
	Data Record 3: Drug mappings	
*Files*	2 files, for the drug ingredient list and drug class list	
*File:*	Drug ingredient mappings	*drug_mappings_ingredients.tsv*
*Columns*	Drug ingredient name	*drug_name*
	RxNORM CUI number	*rxcui*
	ATC code	*atc_code*
	Second-level ATC drug class name (redundant, provided for convenience)	*atc_name*
	Estimated median cost per day	*estimate_drug_cost_per_day*
	UMLS CUI	*UMLS_CUI*
	Drug Bank ID	*DrugBankID*
*File:*	Drug class mappings	*drug_mappings_atc_classes.tsv*
*Columns*	Second-level ATC code	*atc_class*
	Second-level ATC drug class name	*atc_class_name*

**Table 3:** Common 3-drug combinations most overrepresented prior to ED visits.

**Rank**	**Drug combination**	**Relative risk**		
**1**	acetaminophen	oxycodone	prochlorperazine	3.6
**2**	acetaminophen	enoxaparin	oxycodone	3.6
**3**	acetaminophen	hydrocodone	prochlorperazine	3.5
**4**	acetaminophen	enoxaparin	warfarin	3.5
**5**	acetaminophen	enoxaparin	hydrocodone	3.4
**6**	acetaminophen	dexamethasone	oxycodone	3.1
**7**	acetaminophen	levofloxacin	oxycodone	2.8
**8**	acetaminophen	ciprofloxacin	phenazopyridine	2.7
**9**	ondansetron	sulfamethoxazole	trimethoprim	2.7
**10**	acetaminophen	codeine	sulfamethoxazole	2.6
**11**	acetaminophen	levofloxacin	metoprolol	2.6
**12**	levofloxacin	sulfamethoxazole	trimethoprim	2.6
**13**	acetaminophen	codeine	trimethoprim	2.6
**14**	acetaminophen	ciprofloxacin	sulfamethoxazole	2.6
**15**	amoxicillin	clavulanate	ondansetron	2.6
Patients prescribed these common 3-drug combinations visit the ED at rates approximately 3-fold higher than the general population. Overrepresentation is calculated by comparing the incidence of 3-drug combination exposures in the 30-day window prior to ED visits (based on only the first ED visit per patient) to their overall incidence, as recorded in Data Record 1. This table includes only common 3-drug combinations, with greater than 5000 occurrences in the database.				

**Table 4:** Summary of the most common and most overrepresented drug ingredient co-exposures with metformin and oxycodone.

**Rank**	**Drug ingredient**	**P(co-exposure | exposure)**	**Observed/Expected Incidence**
*Metformin co-exposures, top 5, ranked by incidence:*			
1	hydrochlorothiazide	0.26	2.2
2	lisinopril	0.25	3.0
3	simvastatin	0.22	2.7
4	atorvastatin	0.15	2.6
5	amlodipine	0.13	2.2
*Metformin co-exposures, top 5, ranked by overrepresentation:*			
1	glyburide	0.10	12.6
2	saxagliptin	0.02	11.9
3	sitagliptin	0.11	11.7
4	rosiglitazone	0.03	11.2
5	dapaglifozin	<0.01	10.8
*Oxycodone co-exposures, top 5, ranked by incidence:*			
1	acetaminophen	0.78	11.1
2	hydrocodone	0.16	3.1
3	hydrochlorothiazide	0.11	0.9
4	alprazolam	0.10	3.7
5	zolpidem	0.09	3.1
*Oxycodone co-exposures, top 5, ranked by overrepresentation:*			
1	methylnaltrexone	<0.01	23.7
2	oxymorphone	0.01	22.1
3	fentanyl	0.04	16.7
4	morphine	0.04	15.5
5	methadone	0.02	14.6
